# Monitoring complications of botulinum toxin in community practice: A pharmacological study

**DOI:** 10.6026/973206300223718

**Published:** 2026-06-30

**Authors:** Ambili Remesh, Taskeen Kaur Sethi, Sree Ram Subba Reddy Gudimetla, J. Dhanuja Rani, Debanti Giri, Rahul Tiwari, Heena Dixit Tiwari, Afroz Kalmee Syed

**Affiliations:** 1 Department of Pharmacology, Sree Uthradom Thirunal Academy of Medical Sciences, Thiruvananthapuram, Kerala, India; 2 Department of Oral and Maxillofacial Surgery, Luxmibai Dental College, Patiala, Punjab, India; 3 Department of Oral and Maxillofacial Surgeon, Sree Mithra Dental and MaxFace Specialists, Tanuku andhra Pradesh, India; 4 Department of Oral & Maxillofacial Pathology, Government Dental College and Research Institute, Ballari, Karnataka, India; 5 Department of Oral Medicine and Radiology, Dr. R Ahmed Dental College and Hospital, Kolkata, West Bengal, India; 6 Department of Dental Research Cell, Dr. D. Y. Patil Dental College and Hospital, Dr. D. Y. Patil Vidyapeeth (Deemed to be University),Pimpri, Pune 411018, Maharashtra, India; 7 Department of Blood Cell, Commissionerate of Health and Family Welfare, Government of Telangana, Hyderabad, India; 8 Department of Oral and Maxillofacial Pathology, Scientific Medical Writer, Writing and Publications, Tenali andhra Pradesh, India

**Keywords:** Botulinum toxin, adverse effects, pharmacovigilance, cosmetic procedures, drug safety

## Abstract

Botulinum toxin is widely used for cosmetic wrinkle reduction, hyperhidrosis, chronic migraine and facial muscle disorders, but concerns persist regarding adverse effects related to injection technique, toxin diffusion and practitioner variability. Therefore, it is of interest to evaluate the complication rates of community-based botulinum toxin therapy and characterize the clinical presentations of its associated adverse effects. A prospective pharmacological observational study was conducted on 120 patients receiving botulinum toxin type A injections. Majority of complications were mild and transient, with injection-site pain and bruising representing the most common local adverse effects. Thus, data shows the botulinum toxin injections performed by trained practitioners have a favorable safety profile in community practice.

## Background:

Botulinum toxin is a potent neurotoxin derived from Clostridium botulinum that inhibits acetylcholine release at the neuromuscular junction, resulting in temporary muscle relaxation. This pharmacological action has led to its widespread use in both therapeutic and aesthetic medicine. Currently, botulinum toxin is commonly utilized for conditions such as facial wrinkles, hyperhidrosis, spasticity, migraine and various neuromuscular disorders [1]. The popularity of minimally invasive aesthetic procedures has increased dramatically over the past decade. Botulinum toxin injections have become one of the most frequently performed cosmetic procedures worldwide due to their effectiveness, rapid results and relatively low complication rates [2]. Despite its favorable safety profile, adverse effects can occur when injections are performed incorrectly, when inappropriate doses are administered, or when toxin diffusion affects adjacent muscles [3]. Reported complications associated with botulinum toxin therapy range from mild and transient local reactions to more significant functional disturbances. Common local reactions include pain, bruising, erythema and swelling at the injection site. Functional complications such as eyelid ptosis, facial asymmetry and transient muscle weakness may also occur due to unintended toxin spread [4]. Although severe complications are rare, they may include dysphagia, visual disturbances and systemic muscle weakness in susceptible individuals [5]. The increasing use of botulinum toxin in community clinics has raised concerns regarding monitoring and reporting of complications. Unlike hospital settings, community practices may have variable levels of practitioner experience and inconsistent pharmacovigilance systems. Consequently, some adverse reactions may go undocumented or unreported [6]. Recent literature emphasizes the importance of monitoring safety outcomes associated with cosmetic procedures in real-world clinical settings. Identifying patterns of adverse events can help improve injection techniques, optimize dosing strategies and enhance patient counseling before treatment [7]. Several factors influence the likelihood of complications, including injection technique, anatomical knowledge, dosage, dilution methods and practitioner expertise. Patient-related factors such as age, underlying medical conditions and previous aesthetic procedures may also contribute to complication risk [8]. Continuous monitoring and documentation of adverse events are therefore essential for maintaining patient safety and improving treatment outcomes. Community-based pharmacological surveillance can provide valuable insights into real-world safety profiles of widely used injectable agents [9, 10]. Therefore, it is of interest to evaluate and monitor complications associated with botulinum toxin injections in community practice and to analyze the clinical characteristics of these adverse events.

## Materials and Methods:

A prospective observational pharmacological study was conducted in a community-based clinical practice over a period of twelve months. Patients receiving botulinum toxin injections for either therapeutic or aesthetic indications were included in the study. Ethical approval was obtained from the institutional ethics committee and informed consent was obtained from all participants before treatment. Participants aged 18 years or older who underwent botulinum toxin type A injections during the study period were eligible for inclusion. Patients with known neuromuscular disorders, hypersensitivity to botulinum toxin, pregnancy, or incomplete follow-up data were excluded from the study. Baseline demographic information including age, gender, indication for treatment and injection sites were recorded. Botulinum toxin injections were administered by trained practitioners using standardized techniques and dosing guidelines based on clinical indication. Common treatment indications included cosmetic wrinkle reduction, hyperhidrosis, chronic migraine management and facial muscle disorders. Injection techniques followed established safety protocols to minimize the risk of complications. Patients were monitored immediately after the procedure and during follow-up visits scheduled at 1 week, 1 month and 3 months after injection. All adverse effects occurring during the follow-up period were recorded. Complications were categorized into two groups: local complications and functional complications. Local complications included injection-site pain, bruising, erythema, swelling and infection. Functional complications included eyelid ptosis, facial asymmetry, dysphagia or muscle weakness resulting from toxin diffusion. Each adverse event was documented using a standardized complication monitoring form. Complication severity was classified as mild, moderate, or severe depending on clinical impact and requirement for medical intervention. Statistical analysis was performed using SPSS version 26. Descriptive statistics including mean, standard deviation, frequency and percentage were calculated. Associations between demographic variables and complication occurrence were analyzed using chi-square testing with a significance level of p < 0.05.

## Results:

A total of 120 patients received botulinum toxin injections during the study period. The majority of patients were female, reflecting the high demand for cosmetic procedures among women. Cosmetic wrinkle treatment represented the most common indication for injection. Complications occurred in a small proportion of patients and were predominantly mild and transient. No severe systemic complications were observed. The demographic characteristics of the study population are summarized in Table 1. The majority of participants were female and belonged to the 31-50 year age group. This distribution reflects the increased demand for cosmetic procedures among middle-aged adults seeking non-surgical facial rejuvenation. Male participants represented a smaller proportion of patients receiving treatment. Overall, the demographic pattern observed in the study is consistent with global trends reported for aesthetic botulinum toxin procedures. Table 2 shows the clinical indications for botulin toxin injections. Cosmetic wrinkle treatment accounted for the majority of procedures performed in this study. Therapeutic indications such as hyperhidrosis and migraine management were also observed but occurred less frequently. Facial dystonia represented the least common indication. These findings highlight the increasing use of botulinum toxin for aesthetic purposes in community-based clinical practice. Table 3 presents the local complications observed following botulinum toxin injections. Injection-site pain was the most commonly reported adverse effect, followed by bruising and swelling. Erythema was observed in a smaller number of patients. All local complications were mild and resolved spontaneously within a few days without requiring additional treatment. No cases of injection-site infection were recorded during the follow-up period. Functional complications associated with botulinum toxin injections are presented in Table 4. Eyelid ptosis was the most frequently observed functional complication but occurred in a small proportion of patients. Facial asymmetry and transient muscle weakness were rare events and resolved without long-term effects. No cases of dysphagia or systemic toxicity were reported. These findings confirm the relatively low risk of serious complications when injections are performed by trained practitioners.

## Discussion:

Botulinum toxin injections have become one of the most widely performed minimally invasive procedures in both therapeutic and cosmetic medicine. The results of the present study demonstrate that complications associated with botulinum toxin injections in community practice are generally mild and transient. Local adverse effects such as injection-site pain, bruising and swelling were the most commonly observed complications. Similar findings have been reported in recent clinical reviews evaluating the safety profile of botulinum toxin therapy [11]. The pharmacological mechanism of botulinum toxin involves inhibition of acetylcholine release at neuromuscular junctions, resulting in temporary muscle paralysis. While this mechanism provides therapeutic benefit in a wide range of medical and aesthetic conditions, it also explains the occurrence of functional complications when toxin diffusion affects adjacent muscles [12]. Local injection-site reactions were the most frequent complications observed in the present study. These reactions are typically associated with needle trauma or minor vascular injury during injection. Previous studies evaluating cosmetic botulinum toxin procedures have also reported injection-site pain and bruising as the most common adverse events [13]. Eyelid ptosis was the most frequently observed functional complication in the present study, although it occurred in only a small proportion of patients. This complication occurs when botulinum toxin diffuses into the levator palpebrae superioris muscle. Literature suggests that the incidence of ptosis following cosmetic botulinum toxin injections generally ranges between 1% and 5% [14]. Facial asymmetry and transient muscle weakness were observed in a limited number of patients. These complications are typically related to uneven toxin distribution or incorrect injection depth. Studies evaluating injection technique have emphasized the importance of anatomical knowledge and precise dosing in preventing such complications [15]. The absence of severe systemic complications in this study supports the favorable safety profile of botulinum toxin when administered by trained practitioners. Although rare, systemic adverse effects such as dysphagia or generalized muscle weakness have been described in the literature, particularly when large doses are used or when toxin spreads beyond the intended injection site [16]. Monitoring adverse events in real-world clinical settings is essential for improving patient safety. Pharmacovigilance studies have highlighted the importance of systematic reporting of complications associated with aesthetic procedures. Community-based monitoring systems can help identify emerging patterns of adverse reactions and guide improvements in clinical practice [17]. Another important factor influencing complication rates is practitioner experience. Adequate training in facial anatomy, injection techniques and dosing protocols significantly reduces the risk of complications. Clinical guidelines emphasize the importance of practitioner education and adherence to standardized treatment protocols [18]. Patient-related factors may also influence complication risk. Age, comorbid conditions, previous cosmetic procedures and individual anatomical variations can affect toxin distribution and treatment outcomes. Careful patient selection and thorough pre-treatment evaluation are therefore essential components of safe clinical practice [19]. Overall, the findings of this study confirm that botulinum toxin injections performed in community practice are generally safe and associated with a low incidence of significant complications. Continuous monitoring of adverse events and adherence to safe injection practices remain essential for maintaining optimal patient outcomes [20].

## Conclusion:

Botulinum toxin injections are generally safe procedures when performed by trained practitioners in community practice. Most complications are mild, transient and limited to local injection-site reactions. Structured monitoring and pharmacovigilance systems can further improve patient safety and reduce the risk of complications.

## Advancement to knowledge:

This study provides real-world pharmacovigilance data on botulinum toxin-related complications in community practice and emphasizes the need for structured adverse-event monitoring systems to improve patient safety, practitioner awareness and early identification of injection-related complications.

## Figures and Tables

**Table 1:** Demographic characteristics

**Variable**	**Frequency**	**Percentage**
Female	86	71.7
Male	34	28.3
Age 18-30	22	18.3
Age 31-50	64	53.3
Age>50	34	28.4

**Table 2:** Indications for botulinum toxin injection

**Indication**	**Frequency**	**Percentage**
Cosmetic wrinkle treatment	72	60
Hyperhidrosis	22	18
Chronic migraine	16	13
Facial dystonia	10	9

**Table 3:** Local complications

**Complication**	**Frequency**	**Percentage**
Pain	18	15
Bruising	14	11.7
Swelling	10	8.3
Erythema	6	5

**Table 4:** Functional complications

**Complication**	**Frequency**	**Percentage**
Eyelid ptosis	4	3.3
Facial asymmetry	3	2.5
Muscle weakness	2	1.7
Dysphagia	0	0
